# Consensus queries in ligand-based virtual screening experiments

**DOI:** 10.1186/s13321-017-0248-5

**Published:** 2017-11-28

**Authors:** Francois Berenger, Oanh Vu, Jens Meiler

**Affiliations:** 1 Department of Chemistry, Vanderbilt University, Nashville, TN USA; 2 Division of System Cohort, Medical Institute of Bioregulation, Kyushu University, Fukuoka, Japan

**Keywords:** Similarity search, Several bioactives, Consensus query, Ligand-based virtual screening (LBVS), Chemical fingerprint, Potency scaling, MACCS, ECFP4, MOLPRINT2D, Tanimoto score

## Abstract

**Background:**

In ligand-based virtual screening experiments, a known active ligand is used in similarity searches to find putative active compounds for the same protein target. When there are several known active molecules, screening using all of them is more powerful than screening using a single ligand. A consensus query can be created by either screening serially with different ligands before merging the obtained similarity scores, or by combining the molecular descriptors (i.e. chemical fingerprints) of those ligands.

**Results:**

We report on the discriminative power and speed of several consensus methods, on two datasets only made of experimentally verified molecules. The two datasets contain a total of 19 protein targets, 3776 known active and ~ 2 × 10^6^ inactive molecules. Three chemical fingerprints are investigated: MACCS 166 bits, ECFP4 2048 bits and an unfolded version of MOLPRINT2D. Four different consensus policies and five consensus sizes were benchmarked.

**Conclusions:**

The best consensus method is to rank candidate molecules using the maximum score obtained by each candidate molecule versus all known actives. When the number of actives used is small, the same screening performance can be approached by a consensus fingerprint. However, if the computational exploration of the chemical space is limited by speed (i.e. throughput), a consensus fingerprint allows to outperform this consensus of scores.

## Background

Similarity searches help expand the collection of known actives in the early stages of a drug discovery project. Interestingly, similarity searches do not require a diverse collection of active and inactive molecules prior to be used. Sometimes, in a ligand-based virtual screening (LBVS) campaign, only a limited number of active compounds is known. Such compounds could be found from the scientific literature, patent searches or a moderately successful structure-based virtual screen followed by wet-lab testing. This data scarcity might render standard machine learning algorithms inapplicable. Indeed, most Quantitative Structure Activity Relationship (QSAR) methods are data hungry. While expert machine learning users may benefit from recent developments [1, 2], most users will be left in front of several questions: (a) how many actives are needed to create a powerful classifier, (b) which chemical fingerprint should be used to encode those actives, and (c) what is the best way to combine those fingerprints.

Similarity searches that exploit the chemical similarity principle [3] are some of the earliest techniques developed in chemoinformatics [4, 5]. When several ligands are known for a given protein target, they can be used simultaneously to better find novel, putative active molecules.

This study measures the performance and speed of several ways to combine the knowledge about known actives. We investigate the effect of the fingerprint choice, the number of actives and the method used to combine fingerprints. Compared to most previous studies, our datasets are only made of experimentally verified molecules. We also evaluate the effect of scoring speed in CPU-bounded experiments, to show a potential use when screening immense virtual chemical libraries. We finally discuss which combination of fingerprint, consensus size and consensus method gives the best performance or should be avoided.

Using multiple bioactive reference structures has been studied by several authors [6–14]. Shemetulskis et al. [6] have created modal fingerprints, where a bit is set in the consensus query if it is set in a given percentage (the mode) of known active molecules. Xue et al.  [8] have used all bits consistently set in compounds of a same activity class (called consensus bit patterns) and scale factors for those bits to modify the Tanimoto score. This approach is called fingerprint scaling. It increases the probability of finding active molecules by virtual screening. Wang and Bajorath [13] have used bit silencing in MACCS fingerprints to create a bit-position-dependent weight vector. This weight vector modifies the Tanimoto coefficient in order to derive compound-class-directed similarity metrics that improve virtual screening performance compared to conventional Tanimoto searches. Hert et al. [9] have compared merging several fingerprints into a single combined fingerprint, applying data fusion (merging of scores) to the similarity rankings of single queries and approximated substructure searches. Hert et al. found that merging similarity scores and using binary kernel discrimination are the most powerful techniques. Later, Whittle [11] confirmed that fusing similarity scores using the maximum score rule (or the minimum rank if scores are not available) is one of the most powerful strategies. For a recent study of data fusion methods with fragment-like molecules, see Schultes 2015 [14].

Ligand-based virtual screening methods have also been extended to take into account the different potency levels of known actives [10, 12]. This potency scaling technique allows to bias the search space towards the detection of increasingly potent hits [10]. To bias the search, a logarithmic weighting scheme based on IC50 1values was introduced. Let $q_i$ be a known active molecule. $q_i$ is assigned a weight $w_i$ given it’s IC50 value (IC50_qi_) and the IC50 value of the least active molecule (IC50_min_):

1 $$\begin{aligned} w_i = log(\mathrm {IC50_{min}}) - log(\mathrm {IC50}_{q_i}) + 1.0 \end{aligned}$$

This logarithmic weighting scheme ensures linear scaling over the entire potency range and attributes a weight of one to the least active molecule [10]. In Vogt and Bajorath [12], the same weighting scheme is used to bias two distinct virtual screening algorithms and applied to a high-throughput screening (HTS) data set of cathepsin B inhibitors. The authors observed that using multiple reference compounds and potency scaling allows to direct the search towards detection of more potent database hits.

The pharmacologically relevant chemical space is immense. A study [15] estimates its size in the order of $10^{33}$ molecules, with up to 36 heavy atoms each. While the database of commercially-available compounds ZINC15 [16] totals $335 \times 10^6$ compounds as of May 2017, there are virtual chemical libraries with more than $166 \times 10^9$ molecules [17, 18]. Hence, one can imagine scenarios where the speed of a virtual screen has its importance.

## Methods

In this study, only fully automatic methods are investigated on 2D fingerprints. None of these methods requires fitting to a training set. The investigated methods are all parameter-free. Implicit parameters, if any, are detailed.

### Datasets

Several protein targets coming from two distinct datasets were used. None of those datasets contain any decoy (i.e. molecules that have not been experimentally verified as either active or inactive). A decoy is a supposedly inactive, computationally engineered [19] or randomly chosen [8] molecule. Hence, in this study there is no risk that a decoy creation protocol can be reverse engineered by any of the evaluated methods. Also, some of the datasets used are real world examples since they come from HTS campaigns.

**Table 1:** The NRLiSt subset with IC50 data for all actives

Protein target	# Actives	# Inactives
AR−	179	179
ERα-	74	434
ERα+	102	132
ERβ+	70	70
GR−	204	295
GR+	74	369
PR−	269	269
PR+	74	531
RARα−	41	133
RXRα−	114	210

‘+’ after a target name means actives (resp. inactives) have an agonist (resp. antagonist) effect. ‘−’ after a target name means the opposite

Our first dataset is the manually curated nuclear receptors ligands and structures benchmarking database [20] (NRLiSt BDB^2^). The NRLiSt is an exhaustive, NR-focused, benchmarking database. The original NRLiSt contains 9905 ligands and 339 protein targets. However, for the specific needs of this study, the NRLiSt was further filtered in order to contain only protein targets for which there are at least 40 known actives and at least the same amount (or more) of tested inactives. Furthermore, only active molecules with an IC50 value are accounted for. A summary of this dataset is given in Table 1.

**Table 2:** The nine HTS datasets with their PubChem Summary Assay ID (SAID)

Protein target class	SAID	# Actives	# Inactives
GPCR	435008	233	217,925
	1798	187	61,646
	435034	362	61,394
Ion channel	1843	172	301,321
	2258	213	302,192
	463087	703	100,172
Transporter	488997	252	302,054
Kinase inhibitor	2689	172	319,620
Enzyme	485290	281	341,084

The second dataset, MLQSAR,^3^ is a compilation of validated PubChem High Throughput Screens [21]. PubChem provides libraries of small molecules that have been tested in HTS experiments. This dataset focuses on experiments with a single well-defined and pharmaceutically relevant protein target. A target is only retained if it has at least 150 confirmed active compounds. A summary of MLQSAR is given in Table 2. For several targets, active molecules just have a flag and no IC50 data. There are only two targets (PubChem SAID 485290 and 435,008) for which activity values span several orders of magnitude, while this is the case for all NRLIST targets.

### Fingerprints

In this study, three different fingerprints were used. The MACCS 166 bits fingerprint as provided by Open Babel [22]. The ECFP4 2048 bits fingerprint as provided by Rdkit [23, 24] and an unfolded MOLPRINT2D [25, 26] implementation, referred to as UMOP2D in the text. MOLPRINT2D descriptors encode atom environment based on SYBYL atom types (i.e. atom type and hybridization state) derived from the molecular graph. Only heavy atoms and their connected neighbors up to a distance of two bonds are considered by this fingerprint. MOLPRINT2D will be available in the upcoming version of the Bio Chemical Library [27]. The goal of this study is not to compare the power of individual fingerprints; see Sastry [28] for an extensive comparison.

### Chemical similarity

To fairly compare methods during experiments, the Tanimoto score is used consistently. Given two binary fingerprints ${\mathbb {A}}$ and ${\mathbb {B}}$ of equal length:

2 $$\begin{aligned} Tani({\mathbb {A}},{\mathbb {B}})=\frac{|{\mathbb {A}} \cap {\mathbb {B}}|}{|{\mathbb {A}} \cup {\mathbb {B}}|} \end{aligned}$$

Given two fingerprints ${\mathbb {X}}$ and ${\mathbb {Y}}$ encoded as vectors of floats of length N:

3 $$\begin{aligned} Tani({\mathbb {X}},{\mathbb {Y}}) = \frac{\sum _{i=1}^Nx_i y_i}{\sum _{i=1}^N{x_i^2 + y_i^2 - x_i y_i}} \end{aligned}$$

### Performance metrics and curves

To assess overall classifier performance, the Area Under the receiver operating characteristic Curve (AUC) is used. To measure early retrieval performance, the Power Metric at 10% ($\hbox {PM}_{10\%}$) is used [29]. The power metric is a function of the True Positive Rate (TPR) and False Positive Rate (FPR) at a given threshold. The power metric is statistically robust to variations in the threshold and ratio of active compounds over total number of compounds in a dataset. At the same time, the power metric is also sensitive to variations in model quality.

4 $$\begin{aligned} PM_{x\%} = \frac{TPR_{x\%}}{TPR_{x\%} + FPR_{x\%}} \end{aligned}$$

In some experiments, the accumulated number of actives is drawn. This curve is obtained by walking down a rank-ordered list of compounds (the X axis lists ranks of database molecules) and plotting on the Y axis the number of active molecules encountered so far.

### Consensus policies

In all methods described hereafter, there is no parameter fitting to any of the datasets. Also, no training using known actives and inactives is required prior to applying any of the methods.

**Fig. 1:**
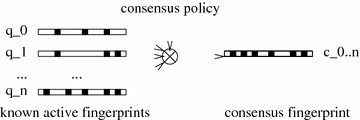
A consensus fingerprint is created by combining the fingerprints of several known active molecules. The way to combine fingerprints is controlled by the consensus policy

A consensus query is formed by the combination of a set of query molecules (also called known actives) while following a consensus policy. The policy specifies how fingerprints are combined (Fig. 1).

Let ${\mathbb {P}}$ be the set of all protein targets. Let *p* be a given protein target ($p \in {\mathbb {P}}$).

Let ${\mathbb {M}}$ be the set of all tested molecules for *p*.

Let *fp*(*m*) be the fingerprint of molecule $m \in {\mathbb {M}}.$

Let ${\mathbb {A}}$ be the set of active molecules on *p* (${\mathbb {A}} \subset {\mathbb {M}}$).

Let ${\mathbb {I}}$ be the set of inactive molecules on *p* (${\mathbb {I}} = {\mathbb {M}} {\setminus} {\mathbb {A}}$).

Let ${\mathbb {Q}}$ be a randomly selected set of actives that will be used to build a consensus query of size *N* (${\mathbb {Q}} \subset {\mathbb {A}} \wedge |{\mathbb {Q}}| = N$). In this study, only two to 20 actives are used to create a consensus query ($2 \le N \le 20$).

Let ${\mathbb {C}} = {\mathbb {M}} \setminus {\mathbb {Q}}$ be the set of candidate molecules for *p*; sometimes referred to as the “database” of molecules to screen.

During a retrospective ligand-based virtual screening experiment, the active or inactive status of a molecule $c_i \in {\mathbb {C}}$ is ignored, until the final computation of a performance metric or curve is triggered.

We call query $q_i$ a molecule randomly drawn from ${\mathbb {Q}}$. Let $score(q_i, c_j) \mid q_i \in \mathbb {Q} \wedge c_j \in \mathbb {C}$ be the Tanimoto score of the fingerprint of the query molecule at index *i* with the fingerprint of the candidate molecule at index *j*. If *x* is a consensus query of fingerprint type, writing $score(x, c_j)$ is also valid.

The list of policies described hereafter are: single, pessimist, optimist, realist and knowledgeable. Policies are sometimes abbreviated using their first four letter.

Let $\mathbb {O}$ be the set of all consensus policies:

$$\mathbb {O} = \{Sing,Oppo,Pess,Opti,Real,Know\}$$.

Let $cscore(o, \mathbb {Q}, c_i)$ be the consensus query score using policy $o \in \mathbb {O}$ and set of known actives $\mathbb {Q}$ with candidate molecule $c_i.$

#### Single query

In the single policy, each active is used in turn as the query molecule. This policy reproduces the average performance of using a single bioactive molecule as query instead of several.

5 $$\begin{aligned} cscore(Sing, q_i, c_i) = score(fp(q_i), fp(c_i)) \end{aligned}$$

#### Opportunist consensus of scores

The score assigned to a candidate molecule is the maximum score it gets over all query molecules. This consensus query is a set of fingerprints. In the literature [9, 11], this method is classified as a data fusion method and called max of scores or minimum of ranks.

6 $$\begin{aligned} &cons(Oppo, \mathbb {Q}) = \mathbb {Q}\\&cscore(Oppo, \mathbb {Q}, c_i) = \\&max\{score(fp(q_i), fp(c_i)) \forall q_i \in \mathbb {Q}\} \end{aligned}$$

#### Pessimist

The consensus query is the fingerprint resulting from doing a bitwise AND of all query fingerprints. This consensus is a single fingerprint. This is the “consensus bit pattern” from Xue et al. [8].

7 $$\begin{aligned}&x = cons(Pess, \mathbb {Q}) = \cap \{fp(q_i) \forall q_i \in \mathbb {Q}\}\\&cscore(Pess, \mathbb {Q}, c_i) = score(x, fp(c_i)) \end{aligned}$$

#### Optimist consensus of fingerprints

he consensus query is the fingerprint resulting from doing a bit-wise OR of all query fingerprints. This consensus is a single fingerprint.

8 $$\begin{aligned}&x = cons(Opti, \mathbb {Q}) = \cup \{fp(q_i) \forall q_i \in \mathbb {Q}\}\\&cscore(Opti, \mathbb {Q}, c_i) = score(x, fp(c_i)) \end{aligned}$$

#### Realist

 The consensus query is the float vector where each index *i* of the vector contains the probability for bit *i* of being set over all queries. This consensus is a single fingerprint.

9 $$\begin{aligned}&x = cons(Real, \mathbb {Q}) =\\&[p(bit_j = 1) \forall j \in bits(fp(q_i)) \forall q_i \in \mathbb {Q}]\\&cscore(Real, \mathbb {Q}, c_i) = score(x, c_i) \end{aligned}$$

#### Knowledgeable

 This consensus is almost like the realist consensus, except that query molecule fingerprints are potency-scaled (cf. formula 1) prior to being taken into account. This consensus is a single fingerprint.

10 $$\begin{aligned}&\mathbb {Q}_w = potency\_scale(\mathbb {Q})\\&x = cons(Know, \mathbb {Q}_w) =\\&[p(bit_j = 1) \forall j \in bits(fp(q_i)) \forall q_i \in \mathbb {Q}_w]\\&cscore(Know, \mathbb {Q}_w, c_i) = score(x, c_i) \end{aligned}$$

### Software

Our software, called Consent, is written in OCaml. OCaml is a statically-typed functional programming language allowing fast prototyping of scientific software [30].

We release all our software, scripts and datasets as open source. MACCS 166 bits (resp. ECFP4 2048 bits) fingerprints were computed using a small C++ program linked to Open Babel v2.4.1 (resp. RDkit v2015.03.1). MOLPRINT2D unfolded fingerprints are computed by Consent.

Consent can exploit multicore computers thanks to the Parmap [31] library. Some of the dataset preparation, running of experiments and post processing of results were accelerated by PAR [32].

### Experiments

#### Consensus query experiments

The size of the consensus is varied from 2 to 20 actives. Experiments don’t go over 20 actives, because it is imaginable that if more than twenty actives are known for a given target, one might also have many known inactives and could be training a QSAR model. Also, some protein targets of our datasets only have 40 actives, so it is not allowed to use more than half of them to build a consensus query. Active molecules used to build a consensus query are randomly drawn from the actives of the given protein target. Actives used to build a consensus query are also removed from the database to screen. Hence, benchmarks don’t become artificially easier as the consensus size is grown.

On the NRLIST dataset, experiments are repeated at least a hundred times since this dataset is small. On the MLQSAR dataset, experiments are repeated up to 20 times. MLQSAR is quite large and calculating statistics on it is costly. When a performance curve is reported, this curve is the median curve obtained during experiments using the same (protein target, consensus size, consensus policy) experiment configuration triplet. Calculating this median curve is memory intensive, especially for MLQSAR.

#### Speed experiments

Speed experiments were performed on PubChem SAID 485290, which contains 341,365 active and inactive molecules (largest dataset). The virtual screen was run once, then the median throughput (in molecule/s) of the five subsequent runs was computed.

Experiments were performed using a single core of an Intel Xeon CPU at 3.50 GHz on a Linux CentOS v6.8 workstation equipped with 64 GB or RAM.

#### Potency-scaling experiments

In potency scaling experiments, two consensus policies are compared. The database of compounds for a given target is rank ordered several times and the median rank for each active molecule with each consensus policy is recorded.

To compare two consensus policies, active molecules are first ordered by decreasing IC50, then the difference of ranks between the two policies is measured. This allows to compute a delta rank plot. A negative delta rank is a positive outcome: the given active molecule went higher in the rank-ordered list of compounds (i.e. it is found earlier by the virtual screen). A positive delta rank is the opposite negative outcome.

#### CPU-bounded experiments

In CPU-bounded experiments, two consensus policies are compared and the faster policy is allowed to score more molecules. For example, if policy $p_1$ is two times faster than policy $p_2$. Then, $p_2$ will only screen a random half partition of the database while $p_1$ will screen the whole database.

This experiment simulates an *in silico* combinatorial chemistry library enumeration [18, 33–36], where molecules are generated, fingerprinted and scored on the fly. The virtual screen is stopped after some amount of time, not because the immense library enumeration has finished.

## Results

### Effect of the consensus size

#### Discriminative power

**Fig. 2:**
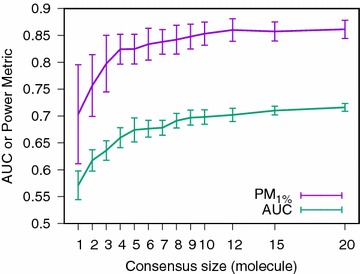
Effect of the consensus size on the consensus query global classification performance (AUC) and early recovery capability ($\hbox {PM}_{1\%}$). Experiment: HTS dataset PubChem SAID 463087, ECFP4 fingerprint and optimist consensus. Values shown are medians over 100 experiments ± 1 median absolute deviation [37]

As the consensus size is growing, the power to discriminate between active and inactive molecules increases.

When looking at the global performance of the classifier (monitored by its AUC) as well as its early recovery capability (monitored by its $\hbox {PM}_{1\%}$ [29]), there is a clear improvement correlated with the growth in consensus size (Fig. 2). On this target, there is no more improvement in early recovery capability once a consensus of size twelve is reached. A bigger consensus only improves the AUC.

#### Speed

**Fig. 3:**
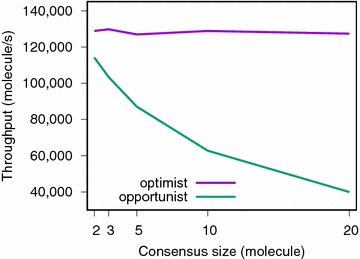
Speed comparison between the opportunist (oppo) and optimist (opti) consensus policies on ECFP4 fingerprints as a function of the consensus size

While a consensus fingerprint query screens at a constant speed of ~ 130,000 molecule/s (Fig. 3), this is not the case for a consensus based on scores.

Figure 3 shows a comparison of speed between the optimist and the opportunist consensus. As the number of known actives used to build the consensus is growing, the opportunist consensus becomes slower.

### Effect of the consensus policy and fingerprint type

#### NRLIST dataset

**Fig. 4:**
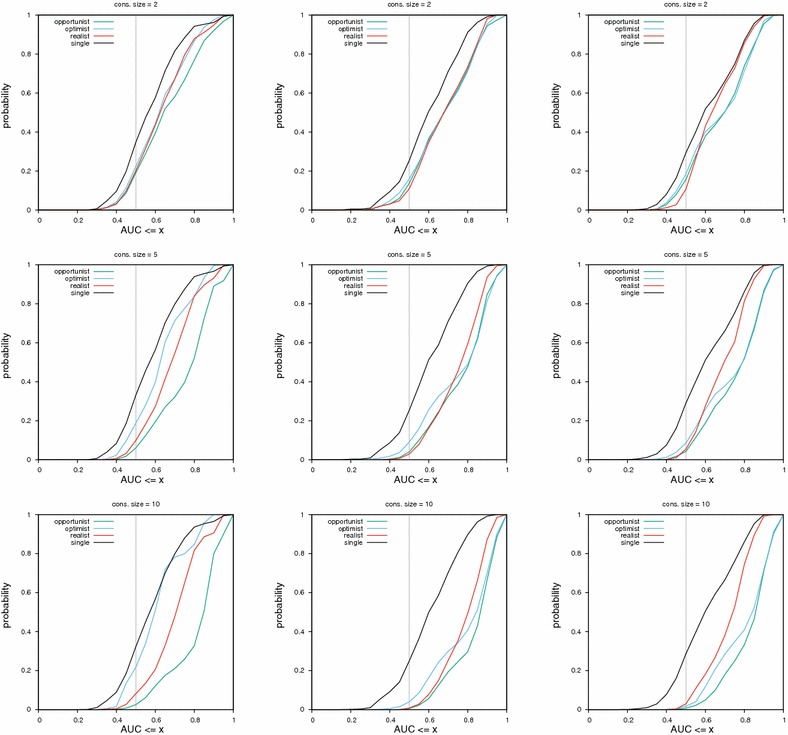
Cumulative distribution functions of AUC values for consensus of sizes two, five and ten. The consensus was built using MACCS fingerprints in the left column, ECFP4 fingerprints in the middle and UMOP2D fingerprints on the right. The lower a curve is, the better the corresponding method. The vertical gray bar at AUC 0.5 allows to find the least random method (lowest curve)

**Fig. 5:**
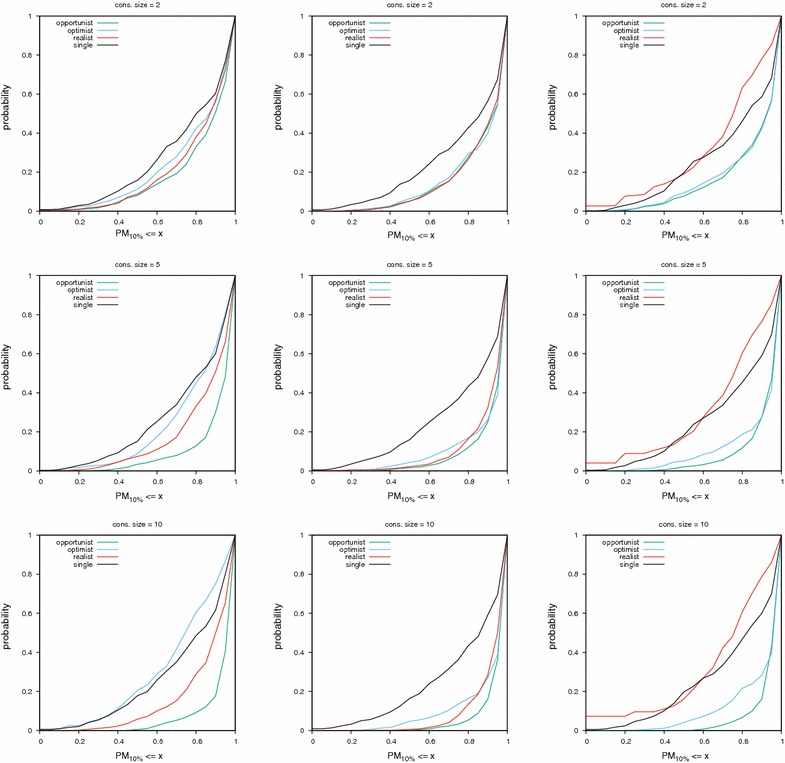
Cumulative distribution functions of $\hbox {PM}_{10\%}$ values for consensus of sizes two, five and ten. The consensus was built using MACCS fingerprints in the left column, ECFP4 fingerprints in the middle and UMOP2D fingerprints on the right. The lower a curve is, the better the corresponding method

On this dataset, results across consensus policies and fingerprints can be seen in Fig. 4.

For MACCS fingerprints, the most efficient policy is the opportunist, followed by the realist consensus. The optimist consensus performs less well than a single query when the AUC reached is greater than 0.85.

For ECFP4 fingerprints, the most efficient policy is also the opportunist one. Then, the realist consensus or the optimist consensus (for AUC ≥ 0.75) are the most powerful. With this fingerprint, all consensus policies have a better performance than a single query, as can be seen in the gap between the black curve and all other curves.

For the UMOP2D fingerprint, the trend is similar than ECFP4. However, the optimist consensus is always better than the realist one and can even outperform the opportunist consensus for PM values ≥ 0.8 (Fig. 5).

The realist consensus is not shown on these AUC plots. Its performance is very similar to the realist consensus but its effect is different and shown later in “Effect of potency-scaling” section.

Across fingerprints, the least random and best performing method is always the opportunist consensus. However, as the consensus size gets smaller, the spread between curves (and hence the performance difference between methods) becomes smaller.

It is interesting to observe that the trend is different for each fingerprint. If something is observed for one fingerprint, it might not hold for other fingerprints.

#### MLQSAR dataset

**Fig. 6:**
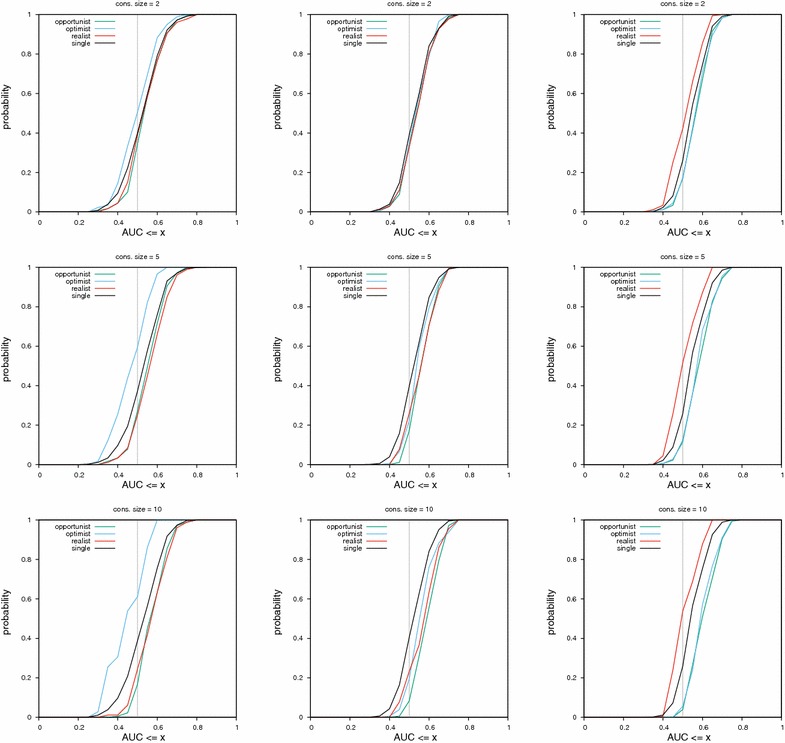
Cumulative distribution functions of AUC values on the MLQSAR dataset. Cf. Fig. 4 for legend details

**Fig. 7:**
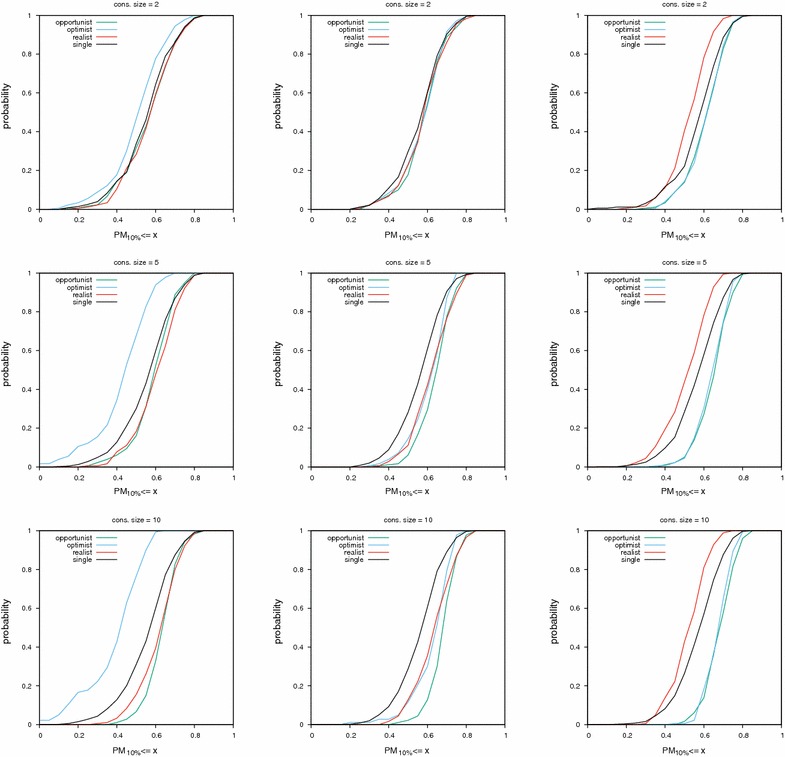
Cumulative distribution functions of $\hbox {PM}_{10\%}$ values on the MLQSAR dataset. Cf. Fig. 5 for legend details

Results on this dataset differ from results on the NRLIST. As a general trend, the spread between curves for different consensus policies is smaller. The biggest difference is that even by looking only at AUC values, the optimist consensus on MACCS fingerprints and the realist consensus on UMOP2D fingerprints are clearly disqualified. They perform worse than a single query. While on the NRLIST, this observation can only be made by looking at CDF curves of PM values.

Experiments on this dataset show that combining the realist policy with the MACCS or ECFP4 fingerprints can outperform the performance of the opportunist consensus (left and middle columns of Figs. 6 and 7, yellow curve under all other curves).

**Table 3:** Median AUC and PM values with their median absolute deviations

Metric	Csize	Policy	MLQSAR dataset			NRLIST dataset		
			MACCS	ECFP4	UMOP2D	MACCS	ECFP4	UMOP2D
AUC	2	Oppo	0.542±0.059^✠▼^	0.548±0.052^✠^	0.571±0.055	0.643±0.119	0.699±0.124	0.696±0.131
		Opti	0.514±0.061^▼^	0.542±0.046^✠◊^	0.564±0.057^◊^	0.624±0.115	0.698±0.129^◊^	0.700± 0.142^◊^
		Real	0.549±0.061^◊^	0.052±0.053^✠◊^	0.518±0.060^▼^	0.632±0.106	0.696±0.119^◊^	0.646±0.105
		Sing	0.544±0.064^◊^	0.530±0.053^◊^	0.540±0.051	0.581±0.100	0.603±0.104	0.598±0.122
	5	Oppo	0.543±0.055	0.530±0.053	0.577±0.057	0.577±0.057	0.814±0.088	0.799±0.992
		Opti	0.460±0.073^▼^	0.536±0.040	0.569±0.051^◊^	0.625±0.092	0.802±0.111	0.802±0.098
		Real	0.569±0.058^◊^	0.562±0.052^◊^	0.496±0.057^▼^	0.675±0.086	0.774±0.087	0.709±0.092
		Sing	0.536±0.065	0.521±0.051	0.536±0.053	0.574±0.100	0.600±0.106	0.598±0.123
	10	Oppo	0.569±0.047	0.586±0.047	0.607±0.051	0.847±0.061	0.873±0.055	0.856±0.060
		Opti	0.435±0.090^▼^	0.554±0.045	0.591±0.043^◊^	0.601±0.078	0.853±0.081	0.841±0.084
		Real	0.571±0.065^◊^	0.578±0.044	0.495±0.053^▼^	0.705±0.078	0.803±0.083	0.738±0.081
		Sing	0.532±0.067	0.525±0.054	0.537±0.049	0.573±0.099	0.597±0.107	0.595±0.125
$\hbox {PM}_{10\%}$	2	Oppo	0.588±0.059^✠^	0.615±0.058	0.615±0.074	0.912±0.088	0.933±0.067	0.933±0.067
		Opti	0.542±0.071^▼^	0.609±0.053^◊^	0.621±0.073^✠◊^	0.875±0.116	0.941±0.059^◊^	0.930±0.070^◊^
		Real	0.588±0.066^✠◊^	0.593±0.074^✠◊^	0.536±0.074^▼^	0.896±0.104^◊^	0.933±0.067^◊^	0.758±0.140
		Sing	0.575±0.077^◊^	0.585±0.075	0.585±0.084	0.826±0.155	0.870±0.130	0.832±0.163
	5	Oppo	0.600±0.058	0.635±0.065	0.651±0.060	0.950±0.050	0.968±0.032	0.959±0.041
		Opti	0.452±0.075^▼^	0.613±0.059^◊^	0.654±0.055^◊^	0.804±0.137^▼^	0.979±0.021	0.969±0.031
		Real	0.612±0.073^◊^	0.623±0.068^◊^	0.523±0.068^▼^	0.899±0.101	0.955±0.045	0.763±0.160^▼^
		Sing	0.569±0.086	0.568±0.079	0.579±0.084	0.817±0.152	0.866±0.130	0.830±0.161
	10	Oppo	0.637±0.047	0.690±0.045	0.688±0.043	0.964±0.036	0.968±0.032	0.956±0.040
		Opti	0.405±0.099^▼^	0.660±0.051	0.674±0.041^◊^	0.726±0.170^▼^	0.973±0.027	0.969±0.031
		Real	0.617±0.063^◊^	0.635±0.074	0.528±0.063^▼^	0.896±0.090	0.951±0.049	0.724±0.146^▼^
		Sing	0.571±0.082	0.576±0.078	0.576±0.079	0.809±0.158	0.862±0.134	0.830±0.155

‘csize’ stands for consensus size. A ‘✠’ indicates that a distribution of peformance metric values is not significantly different from the one of the single policy (Kolmogorov–Smirnov test with p-value $\ge 0.05$). A ‘◊’ indicates that a distribution of peformance metric values is not significantly different from the one of the opportunist policy. A ‘▼’ indicates performance worse than the single policy

Table 3 provides a bird’s-eye view of results shown in Figs. 4, 5, 6 and 7 (same protocol but different experiment).

### Effect of potency-scaling

**Fig. 8:**
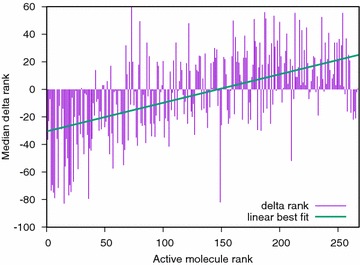
Effect of applying potency scaling (knowledgeable policy) or not (realist policy) to build the consensus. The median change in rank is shown over 1000 experiments for all actives of the NRLIST PR-target (NRLIST target with the most active ligands) and MACCS fingerprints. Active molecules are ranked ordered from most to least potent (left to right)

The effect of potency scaling is that it brings highly active molecules closer to the query but pushes further away less potent molecules (Fig. 8).

**Table 4:** Cases where the knowledgeable consensus policy brings the most active molecules closer to the query compared to another policy

Size	Know verus real	Know versus oppo
10	10/10	8/10
15	10/10	7/10
20	10/10	7/10

Experiment: all NRLIST targets, median change in rank over 500 experiments and ECFP4 fingerprint

In our experiments (Table 4) and in terms of bringing the most active molecules closer to the query, there is a clear advantage of the knowledgeable policy (which is potency scaled) versus the realist one. On the NRLIST, the knowledgeable policy is also better than the opportunist one. However, on the two MLQSAR targets with a wide distribution of potency values, this behavior is observed only once.

#### CPU-bounded experiments

**Fig. 9:**
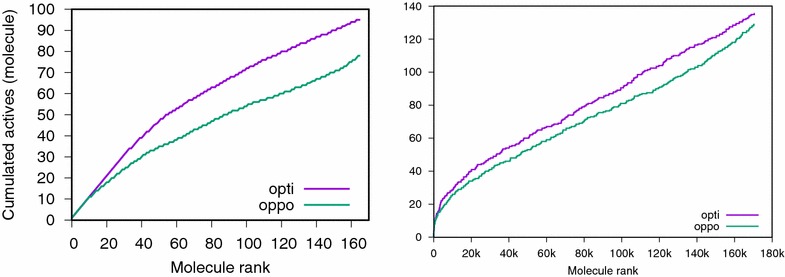
CPU-bounded experiment on the NRLIST AR- target (left) and PubChem SAID 485290 (right)

**Table 5:** Cases where the optimist query outperforms the opportunist one in CPU-bounded experiments

Size	Speedup	MLQSAR	NRLIST
5	1.46	6/9	9/10
10	2.05	7/9	8/10
20	3.19	7/9	8/10

Size: consensus size; speedup: how many times the optimist consensus is faster at scoring than the opportunist consensus. Experiment: compute the accumulated curve of actives on MLQSAR (median curve over 10 experiments) and NRLIST (median curve over 50 experiments). The number of times where the optimist curve dominates the opportunist one is reported

Consensus queries using a single fingerprint can be several times faster than a consensus based on scores (Table 5). For example, a consensus query using only five actives is 1.46 times faster than a consensus of five scores. With 20 actives, it becomes 3.19 times faster.

In theory, a consensus made of N scores could be up to N times slower than a fingerprint consensus. However, our software being optimized, the slowdown is not so high.

In the case where the computational exploration of the chemical space is limited by the speed at which molecules can be scored, there is an advantage at using a consensus query which is faster than a consensus of scores (Fig. 9 and Table 5). In at least six out of nine targets from the MLQSAR dataset, the optimist consensus outperforms the opportunist one in CPU-bounded experiments. On the NRLIST dataset, the same trend is observed in at least eight out of ten targets.

## Discussion

We say a consensus fingerprint degenerates when its performance become worse than that of a single query, either in terms of global classification (AUC) or early retrieval ($\hbox {PM}_{x\%}$). Based on Figs. 4, 5, 6, 7 and Table 3, we give some warnings and recommendations.

It is safe to use the opportunist policy (consensus of scores) for all the fingerprints we tested. Also, five actives are enough to build a consensus query that will perform significantly differently compared to the single policy (Table 3).

In our setting, all consensus policies are safe to use with the ECFP4 fingerprint. We note that the realistic consensus sometimes outperform the opportunist one in terms of early retrieval (middle column in Fig. 7; PM values ≥ 0.7).

The MACCS fingerprint combined with the optimist policy must be avoided. A diverse set of actives sets too many bits in the consensus fingerprint, rendering it non selective (left column in Figs. 5, 6, 7). MACCS fingerprints combined via the realist policy can be used. Their performance approaches the consensus of scores in the HTS datasets (left column in Figs. 6, 7).

The UMOP2D fingerprint combined with the realist policy must also be avoided (right column in Figs. 5, 6, 7). However, UMOP2D fingerprints combined via the optimist policy allow to approach the performance of the opportunist consensus in terms of AUC and PM (right column in Figs. 4, 5, 6, 7).

The pessimist consensus is not used in our study because when molecules are diverse, the number of set bits in the consensus fingerprint becomes too small, so the resulting fingerprint is non selective. This consensus has been used in the past [6, 8], but for series of congeneric molecules while our experiments use diverse and randomly selected actives.

While we don’t completely disregard the knowledgeable policy, it must be used with caution. If the potency values spread several order of magnitudes and the potency measures are of high quality, using this policy might be useful.

## Conclusions

 In this study, the effect of consensus size, consensus policy and chemical fingerprint choice was benchmarked on decoy-free datasets. It is hoped that these results will be predictive of performance in real world applications.

The consensus policies that were extensively benchmarked are: opportunist (a consensus of scores), optimist (a union of fingerprints), realist (an average of fingerprints) and knowledgeable (an average of potency-scaled fingerprints).

Our results confirm the reliability and performance of the consensus of scores (max score/min rank).

A consensus fingerprint allows to rank-order molecules as fast as a regular fingerprint-based similarity search. If the exploration of the chemical space is limited by the speed at which molecules can be scored, an optimist consensus of ECFP4 or UMOP2D fingerprints can outperform a consensus of scores in terms of finding more active molecules.

As a final remark, consensus queries have a few advantages that are worth remembering:

1. They can be used even when the number of active molecules is scarce. This is interesting in the case of compounds found from literature, patent searches or as a followup to a structure-based virtual screening campaign which found only a handful of actives.
2. They can be used even when there is no information available about inactive molecules.
3. They don’t require a training step prior to be used as classifiers.
4. They are so simple that they can be created and used by non machine learning experts.
5. Last but not least, consensus queries can be used as an additional method, perpendicular to other *in silico* approaches, to confirm which molecules to purchase for wet-lab testing.

**Availability and requirements**

- Project Name: Consent
- Project home page:
- https://github.com/UnixJunkie/consent
- Software archives:
- http://meilerlab.org/software or
- https://zenodo.org/record/1006728
- Operating system: Linux, Mac OS X
- Programming language: OCaml
- Other requirements: the OCaml Package Manager (OPAM), Open Babel, RDkit
- Dataset:
- https://data.mendeley.com/datasets/52hjy6vjwb/1
- License: GPL
- Any restrictions to use by non-academics: None

## Footnotes

1 IC50: 50% Inhibitory Concentration. An IC50 value represents the concentration of a drug that is required to inhibit a given biological process by 50% invitro.

2
http://nrlist.drugdesign.fr.

3
http://www.meilerlab.org/qsar_pubchem_benchmark_2012.
